# Decentralisation and Health Services Delivery in 4 Districts in Tanzania: How and Why Does the Use of Decision Space Vary Across Districts?

**DOI:** 10.15171/ijhpm.2018.97

**Published:** 2018-10-27

**Authors:** Ramadhani Kigume, Stephen Maluka

**Affiliations:** ^1^Department of History, Political Science & Development Studies, Dar es Salaam University College of Education, Dar es Salaam, Tanzania.; ^2^Institute of Development Studies, University of Dar es Salaam, Dar es Salaam, Tanzania.

**Keywords:** Decentralisation, Decision Space, Tanzania

## Abstract

**Background:** Decentralisation in the health sector has been promoted in low- and middle-income countries (LMICs) for many years. Inherently, decentralisation grants decision-making space to local level authorities over different functions such as: finance, human resources, service organization, and governance. However, there is paucity of studies which have assessed the actual use of decision-making space by local government officials within the decentralised health system. The objective of this study was to analyse the exercise of decision space across 4 districts in Tanzania and explore why variations exist amongst them.

**Methods:** The study was guided by the decision space framework and relied on interviews and documentary reviews. Interviews were conducted with the national, regional and district level officials; and data were analysed using thematic approach.

**Results:** Decentralisation has provided moderate decision space on the Community Health Fund (CHF), accounting for supplies of medicine, motivation of health workers, additional management techniques and rewarding the formally established health committees as a more effective means of community participation and management. While some districts innovated within a moderate range of choice, others were unaware of the range of choices they could utilise. Leadership skills of key district health managers and local government officials as well as horizontal relationships at the district and local levels were the key factors that accounted for the variations in the use of the decision space across districts.

**Conclusion:** This study concludes that more horizontal sharing of innovations among districts may contribute to more effective service delivery in the districts that did not have active leadership. Additionally, the innovations applied by the best performing districts should be incorporated in the national guidelines. Furthermore, targeted capacity building activities for the district health managers may improve decision-making abilities and in turn improve health system performance.

## Background and Context

Health sector reforms (HSRs) in low- and middle-income countries (LMICs) have been the major policy focus over the last 4 decades. One of the components of the HSRs is decentralisation. Decentralisation is defined as the transfer of authority and power to manage, plan and make decisions from the central government to agencies and actors at lower levels.^1-3^ Four forms of decentralisation are commonly used by scholars; these are deconcentration, delegation, devolution and privatisation.^1^ Whereas deconcentration refers to moving some workload from the central government to subnational levels, delegation refers to the transfer of decision-making as well as administrative authority for well-defined functions to agencies that are not part of the central government; however, the central government retains some level of indirect control. Devolution, on the other hand, involves creation or reinforcement of subnational levels of government that undertake a set of functions from the central government. These subnational levels have a clear legal status with geographically defined boundaries as well as the statutory authority to raise revenue and determine its expenditure.^1^ Similarly, privatization involves the transfer of functions that would otherwise have been performed by the central government to organisations it does not own, and which are independent and outside the central government.^4^

Inherently, decentralisation grants decision-making space to local level authorities over different functions such as: finance, human resources, service organization, access rules and governance.^5-8^ Besides what is written in official documents, the actual exercise of authorities in the decentralised contexts may be different from what is stated in the policies and thus may vary from one district to another.^6-8^ Similarly, decentralised local-level officials may be more or less striving to take full advantage of powers officially granted to them.^9-12^ For example, pro-active local officials may use the available powers to innovate in order to adapt and improve service delivery in their local areas.^9-12^ Others may continue to rely on the centre for direction, resulting in practices that largely emulate pre-decentralised relationships of authority.^9-12^ A recent systematic review on decentralisation and health equity concluded that where decentralisation leads to high level of autonomy between regions, disparities in healthcare access and financing are more inherent unless mediating factors targeted at those inequalities are implemented.^13^ The factors which may result into disparities between regions include the capacities of local level authorities to design and implement health programmes and the responsiveness of health officials to local elected officials.^9-11,14-16^

Recent studies in Tanzania reported that decentralisation has provided authorities with a range of decision‐making space.^17,18^ However, while district health managers had authority in many health systems function areas, limited capacity of the local government in financial resources highly affected their capacity to make use of the available decision-making space.^19^ There is, however, paucity of studies which have examined the variations in the use of decision space granted by the central government across different districts in Tanzania. This study, therefore, not only examines the exercise of decision space but also it compares the utilization of the granted decision-making space across 4 districts; and explores why variations occur between the same.

### Decentralisation Reform in Tanzania

Tanzania has been implementing decentralization-by-devolution (D-by-D) since 1990s.^20,21^ D-by-D focuses on 4 main dimensions: political, financial and administrative decentralization as well as changed central-local relations.^20^ Administrative decentralisation aimed to delink local government authorities’ (LGAs’) staff from their respective ministries, and grant ability to recruit their own staff and establish their own payroll according the needs of each local government. Financial decentralisation intended to enable district councils to have enough discretional powers to collect financial resources through local taxes and other sources of revenues as well as to enable local governments to prepare their own budgets which reflect their own priorities as well as the ability to excise expenditure from the revenue they earn.^20^ The changed central-local relations intend to empower LGAs in decision-making and that the role of line ministries should be on policy-making, supportive and capacity building, monitoring and quality assurance.

Within the current framework of decentralization, the Ministry of Health, Community Development, Gender, Elderly and Children (MoHCDGEC) is responsible for policy development, strategic planning, resource mobilization, and monitoring and evaluation. The LGAs are responsible for operating and managing primary-level health services, while regions supervise LGAs and manage regional hospitals. The MoHCDGEC shares regulatory and accountability functions with the President’s Office for Regional Administration and Local Government (PO-RALG).

Health sector funding comes from 2 main sources: central tax revenue financed by the government of Tanzania general tax revenue; and Development Partners’ (DPs’) support.^22-24^ DPs provide pooled funding both through general budget support (GBS), and the health basket fund (HBF), a form of sector budget support. Tanzania depends on a significant level of DPs support to finance healthcare, while the share of domestic tax sources is still very low.^22-24^

## Methods

### Theoretical Framework

This study was guided by the decision space framework which was developed by Thomas Bossert.^5^ In this study 4 key functional areas were selected for in-depth analysis of the use of the decision-making space namely: health financing, human resources for health (HRH), medicine and medical supplies, and governance, including community participation. These functional areas were selected because they have widely been used to assess decision space in the context of decentralisation.^5-12,14-16^ As indicated in Table 1, the decision space was determined by assessing the involvement of the central and local level authorities in decision-making.

**Table 1:** Assessment of Decision Space in the Decentralised Health System

**Function area**	**Level of Decision Space**		
	**Narrow**	**Moderate**	** Wide**
**Planning/budgeting/allocation of funds**		
• Local involvement in planning	District health managers mainly refer to central planning norms/targets	District health managers make some local-level adaptations to central planning norms/targets	District health managers mainly include locally defined strategies and targets
• Preparation of regular budget	Budget line items conform mainly to central norms	Local revisions made to budget line items but most allocative decisions made by higher authorities	Local revisions made to budget line items with minimal revisions made by higher authorities
• Allocation and use of funds	The central government mainly set/modify/allocate all funds	District health managers set/modify/allocate some funds	District health managers mainly set/modify/allocate funds
**Human resources**			
• Management of human resources	All HRM functions handled centrally for permanent staff; no ability to contract non-permanent staff	Some HRM functions handled locally for permanent staff (eg, posting, promotion, incentive payment); ability to contract some cadres of non-permanent staff	Most/all functions handled locally (including salary levels); ability to contract most/all cadres of non-permanent staff
**Medicine and medical supplies**	All medicine and medical supplies procured centrally	Some medicine and medical supplies procured locally	District health managers mainly procure medicine and medical supplies
**Governance and community participation**	All governance functions are determined and handled centrally	Some governance functions are determined and handled at the district level	Most governance functions are determined and handled at the district level

Abbreviation: HRM, human resource management. Source: Modified from Bossert et al.^9^

### Study Design and Settings

A descriptive case study design was used.^25^ The study was conducted in 4 districts in Tanzania, namely Iramba (Central Tanzania), Kilolo (Southern Highlands), Rombo (Northern Tanzania), and Liwale (Southern Tanzania). Stratified sampling technique was used to select districts for this study. In the first step, 4 out of 8 health zones were selected purposively taking into consideration variation in geographical representation of the zones. In each zone, one district was randomly selected. In each district, one district hospital was selected purposively for in-depth analysis of the decision-making space. Furthermore, in each district, 2 health centres and 2 dispensaries were randomly selected.

### Data Collection Techniques

The formal range of choices which have been granted to the local authorities was assessed through reviewing key legislations and previous literature. To explore the use of the decision-making space across the 4 districts, we conducted in-depth interviews with a range of key informants. At the national level, the informants included officials in the Ministry of Health and the PO-RALG. At regional and district levels, key informants included Regional Health Management Team (RHMT) and Council Health Management Team (CHMT) members, and health providers. Purposive and snowball sampling techniques were used to identify key informants. The decision space framework informed the interview guides. There was room for probing and delving into topics. Interviews were conducted in Kiswahili language by the first author (RK) from December 2015 to March 2016. Saturation point was reached when no new concepts were identified in successive interviews. As indicated in Tables 2 and 3, a total of 52 interviews were carried out.

**Table 2:** District Level Key Informants

**Category of Respondent**	**Roles in the Decentralisation Process**	**Number of Respondents**			
		**Iramba**	**Kilolo**	**Rombo**	**Liwale**
CHMT	Health planning and budgeting, and implementers of health policies, service delivery, and health quality assurance in the district	3	3	3	3
DEDs	Policy implementers, employers of HRH in the district, revenue generation and decision-makers	1	1	1	1
District Planning Officer	Economic and social planners and policy-makers in the district	1	1	1	1
Health service providers	Deliver health services, planning and budgeting at the health facility	4	4	4	4
**Total**		**9**	**9**	**9**	**9**

Abbreviations: CHMT, Council Health Management Team; DEDs, district executive directors; HRH, human resources for health.

**Table 3:** Regional and National Level Key Informants

**National and Regional Level**	**Role in the Decentralisation Process**	**Number**
Ministry of Health, Community Development Gender, Elderly and Children (former MoHSW)	• Policy-making • Defines priorities for health services • Mobilises resources • Provides technical guidance to organisations involved in service delivery, including districts • Regulates health services delivery	2
PO-RALG (former Prime Minister’s Office for Regional Administration and Local Government)	• Oversees the district and regional health services administratively • Supervises planning, reporting and financial accounting	2
RHMTs	• Oversee the work of regional referral hospitals • Provide technical back up to the district councils in planning and implementation of health plans and budgets • Interpret policies and guidelines and provide support to the districts in the implementation of national policies, guidelines and strategies	12
**Total Key Informants**		**16**

Abbreviations: PO-RALG, President’s Office for Regional Administration and Local Government; MoHSW, Ministry of Health and Social Welfare; RHMTs, Regional Health Management Teams.

### Data Management and Analysis

The study used thematic approach to analyse data.^26^ The following steps were followed in the data analysis. First, the recorded interviews were transcribed verbatim by the first author (RK) and were checked for accuracy by the second author (SM). Second, the interview transcripts were translated from Kiswahili into English by a professional translator and the translations were checked for accuracy by the senior researcher (SM). Third, both authors read between 5 and 10 transcripts to familiarize themselves with the data. Forth, the first author (RK) developed a codebook based on the objectives of the study and conceptual framework. The codebook was shared for review with the senior researcher (SM). Fifth, data were coded manually by the first author (RK) to the identified codes. New codes that emerged during the coding process were added along the way. Saturation was achieved when no more codes emerged from the data. Key themes were then independently identified and organized by level of respondents to facilitate comparisons. Finally, the first author (RK) identified representative quotations for each key theme and were shared with the second author (SM).

## Results

This section presents findings on the use of the decision space granted by the central government to the local government institutions. The findings are organized in terms of the 4 key health system functional areas which were assessed in this study, namely health financing, HRH, medicine and medical supplies and governance, including community participation. The findings are compared across the 4 districts of the study. Relevant examples on the use of decision space by the district health managers, local government officials, and health providers have been provided to reinforce the key points.

### Use of Decision Space in Health Financing

As reported elsewhere, in the framework of decentralisation, districts in Tanzania had authority to mobilise and use locally generated funds to finance different health-related activities.^17^ The Community Health Fund (CHF), the National Health Insurance Fund (NHIF) and user fee were the main sources of local level revenues in the health sector. CHF is a voluntary pre-payment scheme aimed to mobilise financial resources for financing health services in rural areas and informal sector and was rolled out in 2001.^27^ A household contributes an annual payment in order to join CHF membership. The districts have been granted authority to define the annual payment and manage the funds collected from the households.^27^ The annual contribution paid by the household entitles them to a basic package of health services at a primary healthcare facility and hospital level in some districts. The funds collected by the district are doubled by a “matching grant” from the central government. The source of the matching grant is the general government revenue. The NHIF is mandatory for public servants, with other formal sector employees being able to opt into the scheme. User fees in public lower level facilities were introduced in 1993 and typically range from TSh 1000 to TSh 5000 in public primary health facilities. Total funds from CHF, NHIF and user fee revenue are a relatively constant and minimal share of the total budget for health available to LGAs accounting for around 4% of total expenditure.^23^ The subsequent section presents various choices which the districts adopted to mobilise and use local resources within the decentralised health systems.

#### Increasing the Rate of User Fees

Analysis of interviews revealed that in Iramba and Kilolo districts, the district health managers in collaboration with other stakeholders reviewed the amount of user fees in order to attract households to join CHF and discourage out-of-pocket payment. For example, in Iramba District, the amount of user fees increased from TSh 1000 to TSh 3000 and from TSh 1500 to TSh 4000 for the dispensary and the health centre respectively. At the same time, the annual contribution for the CHF was TSh 10 000 per household. Similar finding was reported in Kilolo District where user fees in the health centres were increased from TShs 2000 to TShs 5000. While in Iramba District respondents reported that the idea was initiated by the district health managers, and approved by the Council Health Services Board (CHSB) and other district leaders, in Kilolo District respondents reported that they learned this strategy during their official visit to Iramba District, which was among the best performers in the CHF enrolment in the country.

#### Involving Other Stakeholders in Mobilizing Communities

In Iramba District, district health managers reported that CHF was extensively discussed in various community meetings within the district. The district initiated participatory sensitisation processes involving various departments and stakeholders in the district. Additionally, the district health managers managed to secure high support from the district officials including the District Commissioner (DC) and the District Executive Director (DED). The district health managers highlighted the role played by the DC in increasing uptake of the CHF as exemplified by a district health manager;

*“The major strategy we employed to make CHF a success was to involve politicians. We greatly involved them and they responded positively. They did it as though their responsibility. And that lessened our duties as professionals. This is because politicians know how to play with words”* (IDI with district health manager, Iramba).

Similarly, a Health Manager in Kilolo District reported that they learned this strategy in Iramba District. This significantly helped to increase the enrolment of the CHF. The Health Manager had this to say;

*“Initially, Iringa Region performed very poorly in terms of CHF contribution. We hardly collected 6% of the projected collection. Then the Regional Administrative Secretary instructed us to go to Iramba District, Singida Region, which had reached 70% enrolment. Three of us in the CHMT and another one from the office of the Regional Medical Officer (RMO) went to Iramba District. We learnt their slogan that ‘One chick CHF the whole year’. This helped us to design a theme through which to mobilize the community to join the CHF”* (IDI with a district health manager, Kilolo).

Furthermore, in Liwale District, Health Managers and local level respondents reported that involving politicians at the district and local levels brought significant improvement in the CHF enrolment in the district. One of the Health Managers narrated thus:

*“We have greatly succeeded to increase the number of CHF members after involving politicians from district to village levels. For example, last year we emerged the second best performer nationwide, and we received a prize from the Minister for Health”* (IDI with district health manager, Liwale).

#### Expansion of Benefit Package for the Community Health Fund

Interview data indicated that in 3 districts (Kilolo, Rombo, and Liwale), the CHF benefits were limited to one primary healthcare facility (dispensary or health centre) where the households had registered. This was normally the facility which was closest to the residence of the member. This, according to our respondents, helped making the scheme popular. However, in Iramba District, CHF members were given wider benefit packages and could get healthcare services in any public primary health facility within the district. CHF members were also entitled to some secondary-level services although they were limited to the outpatient section. Respondents reported that expansion of benefit packages motivated many households to join CHF scheme in Iramba District.

#### Limiting the Rate of Exemptions

All district level respondents and majority of health providers reported that the Iramba District managed to convince the village councils to pay for the elders and the poor who were exempted from paying use fees. According to the CHF policy, granting exemptions to the poor and other vulnerable groups is within the mandate of the district.^6^ However, the exemptions are not reimbursed by the central government. District level respondents reported that, in Iramba District, the elderly above 60 years were grouped by 10s to form households and were issued with CHF cards. The cards were purchased by the village governments. This approach was also applied for students in secondary schools.

This approach was also reported to have been successfully used in Liwale District. All district and regional level respondents reported that in the last financial year, Liwale District was the best performer in the CHF enrolment in Lindi Region. The ability of the village government to pay for the exempted groups increased the number of CHF members thereby enabling the district to get a larger share of the matching grant from the central government. The District Health Manager elaborated that:

*“In our District we have succeeded to accommodate vulnerable groups in the CHF. Village governments set aside some money from their revenue for that purpose. Low income households and vulnerable groups are grouped in tens and then given CHF cards which enable them to receive health services in health facilities. This has been so great, and Liwale is the Best Practise in Lindi Region, and we receive good matching fund from the Central Government as a top up”* (IDI with district health manager, Liwale).

In Iramba District, respondents reported that primary school pupils and secondary school students were also grouped into 10s and 5s, respectively. Each primary school pupil was required to contribute TShs 1000 annually (equivalent to US$0.5), and each secondary school student contributed TShs 2000 per year (equivalent to US$1). According to our respondents, this approach became very attractive to both students and parents and it was reported to have contributed significantly to the increased uptake of CHF. One of the Iramba District Health Managers reported that;

*“In our District, we decided to mobilize pupils and students to join the CHF. School children are grouped in tens like ten cells and then each receives a CHF card so that they could be treated at school. Then a good number of school children and parents joined the plan”* (IDI with district health manager, Iramba).

In addition, respondents reported that the district health managers in collaboration with local government officials mobilized individuals in the informal sector like petty traders (Machinga) and motorcyclists (Bodaboda dealers). These groups were also encouraged to form imaginary households of 10s and then joined the CHF. The increased enrolment means that the District Council received a larger share from the matching funds from the central government.

### Use of Decision Space on Drugs and Medical Supplies

Interviews revealed that the 4 districts studied utilised the decision space differently. In Iramba District, the district health managers conducted monitoring and auditing of medicines and medical supplies in the health facilities. The district health managers had realized that the drugs they were receiving from the Medical Stores Department (MSD) were enough. However, the main problem was mismanagement of drugs in the health facilities by health service providers. The district health managers had realized that sometimes drugs designated to the public health facilities were sold in private drug shops. Through frequent auditing, they managed to overcome this problem. One of Iramba District Health Managers illustrated it this way:

*“Initially, there was mismanagement of medicine. Documentation in health facilities was a problem. Health service providers did not record medicines neither in ledgers nor in dispensing books. We decided to carry out inspection in every health facility for drug auditing. We then came to realize that there were a lot of problems; personnel issued medicine indiscriminately. After that the personnel were more careful such that all the supplies received were immediately and carefully recorded and issued out with records”* (IDI with district health manager, Iramba).

Another respondent in Iramba District added that:

*“Accountability in the storage of medicine has helped us a lot. We made it a plan that whenever we visited health facilities, we should visit medical stores; and then see the kinds of medicine that go out faster than others. In order for us to have enough CHF collection, we have to ensure that medicines and medical supplies are constantly available”* (IDI with district health manager, Iramba).

Respondents reported that frequent drug audit also made it possible for the district health managers to identify medicines and medical supplies that were not used at the facility. These drugs were then sent to other needy health facilities. Health service providers were reminded to frequently check their ledgers and communicate immediately to the district health managers about drugs which were available but not needed at their health facility. Another health manager in Iramba District put it this way:

*“Following the auditing of the medicines in the health facilities, it came to our notice that some facilities do have medicines which are not needed while the same medicines are needed in other facilities. We then instructed that if some medicines are not needed in a facility, they should be taken to the DMO’s Office so that they can be directed to the needy facilities. We were very serious about this and we launched a slogan ‘let no medicine expire at your facility; if that happens, it is as good as having a pregnant mother die at your facility’”* (IDI with district health manager, Iramba).

Likewise, the district health managers in Kilolo District reported that they learned the system of drug auditing in Iramba District; and then replicated the system to Kilolo District. The district health managers reported that following this initiative, they had seen significant improvements in the availability of medicines in the health facilities. One of the District Health Managers reported that:

*“We went to Iramba to learn how to audit medicine in our health facilities. After the training, we have directed all in-charges that medicine prescribed to patients should be recorded in ledgers; if a container has 1000 pills, the register should also show 1000 pills. Anyone who breaches this requirement shall face disciplinary measures. So we have succeeded a lot to check irrational use of medicines”* (IDI with district health manager, Kilolo).

However, district health managers reported that in recent years, Iramba District has been facing challenges in maintaining the past achievements in various health system functioning areas including management of medicine and medical supplies as well as sustaining the performance of the CHF. This was attributed to the changes in the top management of the district health system, particularly the District Medical Officer (DMO) and the DC. The former DC was reportedly very influential in promoting CHF. Similarly, the former DMO was reportedly very instrumental in initiating a number of innovations to improve the performance of the district health system. One respondent elaborated it this way:

*“The present DMO is new, and so the supportive supervision we formerly enjoyed is no longer there. We do not understand the DMO, and he too does not understand us. Quite few of us go out for supervision and there isn’t any output since the availability of medicine and medical supply is still a problem, although we buy enough. I can see that the culture of managing supplies has gone down”* (IDI with district health manager, Iramba).

### Use of Decision Space in Human Resources for Health

Within the decentralisation framework, district councils have decision space in the areas of motivation, promotion, and retention of health workers.^18^ The analysis of district health plans and interviews with the district health managers and health service providers revealed that all districts had developed some mechanisms of providing incentives to health providers in order to improve their performance. These incentives included paying subsistence allowances for the newly recruited employees, and giving extra duty and on-call allowances to health service providers when working in extra hours. Other health workers’ retention mechanisms included providing housing to health service providers. This was reported to be widely practiced in all the 4 districts involved in this study.

However, further analysis of the interviews with the district health managers and health service providers revealed significant variations in the use of the decision space with regard to motivation across the 4 districts. For instance, in Iramba District, there was a well-defined schedule of providing extra duty and on-call allowances to health service providers contrary to the other 3 districts. Although district health managers in Liwale and Rombo districts reported that extra duty and on-call allowances were paid to health service providers, interviews with health service providers refuted this claim, adding that it used to be the practice in the past. Respondents reported that in the past they used to receive these allowances but presently they have disappeared completely.

*“Formerly we enjoyed different motivation packages like extra duty and on-call allowances. But that has now stopped; although we still fill in claim forms, we do not know if we shall be paid, and when the payments will be due”* (IDI with health provider, Rombo district).

A health service provider in Liwale had a similar view as he added that;

*“Formerly we were motivated. For example, when we went out to villages for outreach services, we were paid. That also applied to night and extra hours. But nowadays these things have remained a story”* (IDI with health provider, Liwale).

In Iramba District, extra duty allowances were consistently paid to health service providers on a monthly basis. All district health managers reported that all health service providers were constantly paid allowances when they worked for extra hours. This was also confirmed during interviews with health service providers. One respondent pointed out this way:

*“We receive extra duty and on-call allowances. Although sometimes payments are delayed, we at least get paid”* (IDI with health provider, Iramba).

Connected to the above, it was also found that when health providers sent monthly reports to the DMO’s office, they were reimbursed transport costs and paid per diem for at least one night. The district level respondents and health service providers reported that this approach significantly motivated health service providers and lead to timely reporting of the information. A health Manager in Iramba reported that;

*“In our District, if the staff bring monthly reports we give them fare and one per diem. That motivates them to bring the reports every month”* (IDI with district health manager, Iramba).

When asked whether they were receiving any allowances, one of the health providers pointed out this way:

*“We are motivated. For instance, if we do extra duties we receive extra duty allowances and we also receive on-call allowances; although relatively little, at least we get it. Similarly, if an employee takes monthly reports to the District Headquarters, he/she does not use his/her own money; the Council covers that”* (IDI with health provider, Iramba district).

A number of initiatives to motivate health service providers were also reported in Kilolo District. District level respondents reported that in order to improve health workers’ performance and retention, the district health managers designed a number of incentives. First, the District Council through the District Health Department established mechanisms of rewarding health service providers in very remote and hard to reach health facilities. In those facilities, each healthcare provider was given a token allowance of Tshs 100 000 per year. Secondly, the district health managers provided incentive of Tshs 100 000 to health facilities which were able to collect, compile and timely submit to the district health managers accurate and complete health information reports. Similarly, the same amount of money was awarded to the health facilities which scored best in the environmental and cleanliness competitions. These awards and incentives were normally provided during the workers’ annual celebrations, normally held on 1 May each year. A Health Manager from Kilolo District had this to say;

*“In our Council, we decided to motivate those who work in remote health facilities, which we called ‘hard to reach areas’. For every remote facility, we used to pay Ths. 100 000/= to every employee. But also for the best performing facilities in terms of CHF collection and hard environment we paid Tshs. 100 000/= to each. Also in this year’s budget we shall give motorcycles to five (5) villages as motivation for their last year’s performance”* (IDI with district health manager, Kilolo).

A Health manager in the same district added that;

*“In general, in retention mechanism we used to perform well because initially we had a rich reserve of personnel. Currently, 70% of our health facilities have clinicians, and quite few of them fall short. And it is not actually that they are without clinicians; only that they on study leaves. However, if you may have access to the number of employees based on the new 2014–2019 requirement, of course the shortage seems alarming”* (IDI with district health manager, Kilolo).

According to the district health managers, due to the implementation of these initiatives, in 2015 the NHIF awarded Kilolo District a certificate of recognition as one of the best performing districts in human resources management practice in Tanzania. During field work, the first author managed to see the certificate displayed in the DMO’s Office.

In Liwale District, health managers reported that every year they provided incentive to 3 health service providers who were performing extra ordinarily in health facilities. One health provider was chosen from the hospital, one from health centres and one from dispensaries. Like in Kilolo District, the awards were provided during annual workers’ celebrations held on 1 May each year. A heath manager in Liwale District responded this way:

*“The first motivation is for the best workers. We have three winners every year who receive some little packages. Best workers are drawn from the District Hospital, Health Centres and our dispensaries” *(IDI with district health manager, Liwale).

In addition, all district level respondents in Liwale District reported that all newly recruited staff employed at the district hospital were given free accommodation until when they were able to rent houses. This was done in order to attract newly recruited staff and promote retention. During field work, the researcher managed to see some of the houses at the district hospital which were reserved for the newly recruited staff. However, it was evident the houses were more suitable for the employees who had no families.

### Use of Decision Space in the Governance of the District Health System

The analysis of interviews with all district and regional level respondents revealed that in all the 4 districts, health facility governing committees (HFGCs) and CHSBs existed. This implies that there were mechanisms for community participation and governance of the district health services within the framework of decentralisation. Respondents revealed that within the current framework of decentralisation, each council is required to allocate fund in their annual budgets to facilitate the activities of the boards and committees.

However, further analysis of the interviews revealed differences across the 4 districts in terms of the functioning and effectiveness of the committees. While in all the 4 districts health committees faced different challenges which affected their performance, it was evident that in Iramba District, the committees were perceived to perform relatively better compared to Kilolo, Liwale, and Rombo districts. It is thus important to understand the kind of innovations which Iramba District adopted to make these committees perform relatively better. The district respondents and health service providers in Iramba District reported that the district health managers conducted monthly review of HFGCs. The Ministry of Health’s guideline requires that HFGCs meet quarterly.^26^ However, in Iramba the HFGCs met monthly. The additional meetings were held at the discretion of the District Council. One of health managers in Iramba District elaborated that;

*“In our district, these committees meet monthly. Although the guidelines on their institution prescribe meeting quarterly, we decided that they meet monthly so that they have enough time to oversee and supervise service provision in our health facilities. We deliberately did this to enable these committees to supervise CHF and medicines in our”* (IDI with district health manager, Iramba).

Another respondent added that;

*“You know these committees comprise villagers from different areas from the location of the facility. The committees do a great deal in supervising the operation of the facilities. For that matter, we decided that they should meet more often, including once or twice a month, although the guideline suggests they should meet four times in a year”* (Health Worker in Iramba).

Interview data revealed that in all the 4 districts, there was a system of motivating their health committees. The committee members were paid a token allowance during their meetings. The allowances in most of the districts ranged from Tshs 2000 (equivalent to 1$) to Tshs 10 000 (equivalent to 5$). The findings, however, indicated some variations in the 4 districts; while in Iramba District, allowances were consistently paid after every meeting, in Liwale, Kilolo, and Rombo districts members of the committee reported that in most cases, the payment was delayed until the next meeting. In addition, in some cases they were not paid anything. District respondents, health service providers and members of the committees reported that this largely affected the morale of the committee members. A health committee member in Rombo District reported that;

*“As members of Dispensary Committee, we face a lot of challenges. We often come to the Dispensary for different duties. We often spend the whole day here, only to receive nothing in return. We spend our own money and no one refunds us”* (IDI with health committee member, Rombo District).

Another respondent in Liwale District narrated this way:

*“We face a lot of challenges, one of which being lack of any compensation as we attend meetings. For instance, a committee member could come from far away to attend a meeting and does not receive any payment”* (IDI with health committee member, Liwale).

Why Variations in the Use of Decision Space?

This section analyses factors that accounted for the variations in the actual exercise of decision space across the 4 study districts. The key themes that emerged from the analysis of interviews are: skills, knowledge and experience of the district health managers, personalities of the top leaders and support from DPs.

### Skills and Knowledge of District Health Managers

Looking at the qualifications, most of the district health managers had bachelor degrees and some had advanced diploma. In addition, as indicated in Table 4, there were no significant differences in the experiences of the district health managers across the 4 study districts.

**Table 4:** Qualifications and Experiences of the District Health Managers

**District**	**Respondent**	**Age**	**Years of Experience**	**Qualifications**
Liwale	1	36	7	Bachelor of Arts
	2	55	27	Advanced Diploma - Paediatrics
	3	44	44	Bachelor of Medicine
Kilolo	1	58	36	Advanced Diploma - Public health
	2	31	6	Bachelor of Arts
	3	44	7	Bachelor of Pharmacy
Iramba	1	41	14	Bachelor of Medicine
	2	42	20	Advanced Diploma (Mid wifery)
	3	34	5	Advanced Diploma (Paediatrics)
Rombo	1	34	3	Bachelor of Nursing
	2	27	3	Bachelor of Arts
	3	36	2	Bachelor of Pharmacy

Source: Field data, 2017.

### Leadership Skills of the Top Leaders

The relatively better performance of Iramba District was attributed to the personalities of the top leaders at the regional and district level. The DED and the DC were reported to be very influential in promoting CHF. Similarly, the former RMO and DMO were reportedly very instrumental in initiating a number of innovations to improve the performance of the district health system as elaborated by one respondent:

*“Some time back until 2016 we had no serious problems. But towards the end of 2016 to date, we have almost failed. A lot of changes have taken place. The present DMO is new and we do not really understand him. Therefore, even the follow up is now very minimal. We do not understand the DMO, and he too does not understand us. Quite few of us go out for supervision and there isn’t any output since the availability of medicine and medical supply is now beginning to be a problem again, although we buy enough. I can see that the culture of managing supplies has gone down”* (IDI with district health manager, Iramba).

The district health managers underlined the role played by the DC in increasing the CHF uptake. According to our respondents, the DC designed a slogan famously known in Kiswahili as *Kuku mmoja CHF mwaka mzima kwa kaya,* literally meaning “one chick enables a family to pay CHF premium for the whole year.” This slogan encouraged household members to keep chicken that would enable them to pay CHF premium annually as explained by one respondent;

*“The DC used to help us a lot in community meetings because the public tends to trust them more than they trust us. The DC had a famous slogan ‘one chick CHF the whole year’. This was very supportive”* (IDI with district health manager, Iramba).

### Support From Development Partners

In Iramba Districts, respondents highlighted the role played by the World Vision in strengthening leadership and managerial capacity of the regional and district health managers. The capacity strengthening activities were implemented under the project named “Supporting Systems to Improve Nutrition, Maternal New-born and Child Health” (SUSTAIN-MNCH) – a 3-year project funded by the Canadian Government’s Department of Foreign Trade and Development. Among the key system strengthening activities were: training district and regional health managers on planning and budgeting; mentoring of the regional and district health managers on leadership, governance, and supportive supervision; and training and mentoring of regional and district health managers on data entry, validation and analysis using the electronic district health information system software health.

According to our respondents, the teams which received training have continued to provide support to other members who had not received the training from the SUSTAIN project. The CHMT members were of the opinion that the training on planning and budgeting helped increase the capacity of the district and regional health managers and subsequently improve the quality of the district annual health plans. According to our respondent, district annual plans for Singida Region had improved significantly compared to district annual health plans from other districts in Tanzania. It is not surprising that Singida Region received prize from the Ministry of Health and Social Welfare (MoHSW) during the 2013/2014 and 2015/2016 financial year because of high quality of the district annual health plans. The first author (RK) managed to see numerous certificates of outstanding awards displayed in Iramba and at the regional health management offices.

## Discussion

The findings of this study revealed that within the decentralised health systems in Tanzania, some districts were able to make use of the range of choices they were formally granted by the central government while others were unaware of the range of choices they could utilise. In some cases, district health managers and local government officials made effective use of the decision space more than the formal authorities stipulated in the policies. For example, the findings indicated that there were variations on the performance of the CHF between Iramba and other districts. The findings also showed variations in the performance of the health committees and boards. Our findings indicated that while in all the 4 districts health committees were not functioning optimally, in Iramba District, the health committees performed relatively better than in the other districts. It was also evident that there were variations in the performance of the districts on the management of medicines and medical supplies. Furthermore, in the area of HRH management, a number of initiatives were adopted, although there were variations across the districts of the study. For example, the Iramba District health managers introduced the system of providing incentives to health service providers upon submission of monthly reports to the districts. In addition to improving the motivation of health service providers, the initiative was reported to have increased the rate of reporting from health facilities. Like in other areas of the health system performance, other districts in Tanzania, including Kilolo learned good practices from Iramba District and replicated in their districts thereby improving both the motivation of health service providers and the rate of reporting from health facilities.

These findings confirm earlier studies on decentralisation and decision space from other settings which have demonstrated that decentralisation provides a wide range of choice within the formal decision space that is likely to have impacts on the effectiveness of the implementation of decentralisation.^11,12,14-18^ However, the decentralised local-level officials may be more or less striving to take full advantage of powers officially conferred to them.^14-18^ Pro-active local officials may use the available powers to innovate in order to adapt and improve service delivery in their local areas.^14-18^ Others may continue to rely on the centre for direction, resulting in practices that largely emulate pre-decentralised relationships of authority.^14-18^ This implies that the performance of the decentralised local government officials in improving services delivery depends, among other things, on the activeness of the top-leaders at the district and local levels. The theoretical advantages claimed by advocates of the decentralisation reform are not automatically realised at the district and local levels. As Bossert noted it clearly, if local-level officials do not make use of the available decision-making space in their local situations, decentralisation may not automatically achieve the theoretical advantages claimed by advocates of the reform.^17^

The disparities between districts revealed in this study are predictable and this is acceptable in the decentralised health systems as long as the overall gains are higher than under the centralised system. Studies in both low-income and high-income countries have revealed that decentralisation improves the ability of decentralised sub-national levels to address the health needs and preferences of the local population. This inherently results to disparities between jurisdictions in terms of their focus and objectives and may lead into inequities.^13,28^ In order to prevent inequities, it is important for the central coordination to define health system goals and outline the broad frameworks. Equally important, in the decentralised health systems, there is need for mechanisms to redistribute financial resources to mitigate disparity in financing healthcare between districts. The most common mechanisms include a population or need based financial resource transfers and cross subsidisation or equalisation schemes.^29,30^

While the capacities of the decentralised health system managers in terms of human resources and finances have been reported to influence the use of decision-making space in the districts,^15-17,20^ this study underlines the importance of leadership skills and managerial capabilities of the key district health managers and local government officials. While all districts depended financially on the central government and had almost similar levels of human resources and financial capacities, it was the personal initiatives of the key district and regional level managers which contributed to the significant variations in the utilization of the available decision space in the districts. This finding is consistent with the recent studies in Tanzania. For example, Maluka and Bukagile^31^ reported that the personal initiatives of the district level officials contributed significantly in the variations in the uptake of the CHF. Similar findings were reported by Joseph and Maluka in a study on leadership and management practices and their influence on the performance of the CHF in Tanzania.^32^ Another study also attributed the variation in the performance of the HFGCs to the efforts and initiatives of the top-district level leaders.^33^ Furthermore, another study done in Indonesia on decentralization and performance of district health systems also highlighted the importance of personal initiatives of the key district health leaders in getting things done.^34^

The findings of our study suggest that targeted capacity building activities at the district level may improve the decision-making abilities of the decentralized officials and ultimately improve health system performance. The key capacity strengthening skills which can be important include: management and leadership, supportive supervision, and planning and budgeting. Studies elsewhere have reported that institutional capacity building significantly improved the performance of the decentralized health systems.^14,15,35^

Furthermore, our findings underline the importance of horizontal relationships in the decentralized health systems. The district health managers need to work in close collaboration with other district level officials within and outside the health sector. Studies have indicated that the autonomy of any organization depends on the network of relationships at the horizontal level.^36,37^ Therefore, the ability of the decentralized health system organization, in this case, district health management, to utilise the available decision space may be facilitated or hindered by what is possible in their specific local context.^36,37^

Limitations

This study adopted qualitative comparative case study design relying primarily on the in-depth interviews with key informants and analysis of key policy documents. While efforts were made to sample respondents from different levels of decision-making within the framework of decentralised healthcare system in Tanzania, the results of the study are not generalizable to other districts in Tanzania. While the study was conducted in 4 different districts, representing different health zones in Tanzania, the findings presented perspectives of a relatively small number of respondents. However, the in-depth and rich description presented in this thesis provides a valuable contribution to knowledge base on decentralisation, decision space in the context of LMICs. The paper sheds light on how district and local level officials were able or not able to utilize the available decision space to improve the delivery of health services.

## Conclusion

This study concludes that decentralisation inherently creates disparity between different districts in terms of their focus and objectives. Those districts with pro-active managers benefit more from local autonomy than districts with less pro-active managers. More horizontal sharing of innovations among districts may contribute to more effective service delivery in the districts that did not have active leadership. The innovations applied by the excelling district should be incorporated in the national guidelines. Additionally, targeted capacity building activities for the district health managers may improve decision-making abilities and, in turn, improve health system performance. Furthermore, there is a need to forge sustainable partnerships with other key actors in the district. In particular, district health managers need to work in close collaboration with other district level officials within and outside the health sector.

## Ethical issues

This study got approval from the University of Dar es Salaam, Dar es Salaam, Tanzania. The research clearance was then sent to the Regional Administrative Officers in each region of the study for approval in their administrative areas. Verbal informed consent was obtained from all prospective respondents. All respondents were informed of their rights to withdraw from the interview any time they wished. Interviews were audio-recorded after getting consent from the respondents. The transcripts were only accessible to the team members. During the presentation of the findings, no individual identification was attached to the findings. All quotes which were used to illustrate respondents’ and participants’ views had no personal identifiers.

## Competing interests

Authors declare that they have no competing interests.

## Authors’ contributions

RK was involved in conception, design of the study and data collection. SM supervised design of the study and data collection. RK and SM analysed the data. RK drafted the manuscript. All the authors read and approved the final manuscript.

## Authors’ affiliations

^1^Department of History, Political Science & Development Studies, Dar es Salaam University College of Education, Dar es Salaam, Tanzania. ^2^Institute of Development Studies, University of Dar es Salaam, Dar es Salaam, Tanzania.

## Key messages

**Implications for policy makers**

- District health managers need to work in close collaboration with other district level officials within and outside the health sector.
- Involving key district decision makers, including politicians, to mobilise resources at the district and community levels is critically important in effective use of decision-making space.
- Targeted capacity building activities for district health managers may improve decision-making abilities and ultimately improve health system performance.

**Implications for public**

The study provides the public with understanding of the factors for the variations in the use of decision-making space in the context of decentralisation.
